# Measures of depression, generalized anxiety, and posttraumatic stress disorders amongst Yazidi female survivors of ISIS slavery and violence

**DOI:** 10.1186/s13033-020-00412-4

**Published:** 2020-11-10

**Authors:** Perjan Hashim Taha, Shameran Slewa-Younan

**Affiliations:** 1 Psychiatry Unit, College of Medicine, University of Duhok, Duhok, Iraq; 2 School of Medicine, Humanitarian and Development Research Initiative, Translational Health Research Institute, Western Sydney University, Locked Bag 1797, Penrith South DC NSW, Sydney, 1797 Australia; 3 Honorary Senior Research Fellow, Centre for Mental Health, Melbourne School of Population and Global Health, University of Melbourne, Melbourne, Australia

**Keywords:** Yazidi, Enslavement, PTSD, Depression, Anxiety, Suicide

## Abstract

**Background:**

In 2014 the Islamic State of Iraq and Syria (ISIS) undertook a systematic and deliberate campaign against minority groups and non-Sunni Muslim communities. Amongst some of the greatest atrocities were those targeted towards Yazidi communities and in particular their women. The mental health outcomes of those women held in captivity requires investigation. This study sought to examine and compare levels of general psychological distress, depression, generalized anxiety, posttraumatic stress disorder (PTSD) and self-reported suicidal thoughts and behaviors amongst Yazidi women held in captivity compared with those without such experiences.

**Method:**

Between January to May 2019, a total 348 Yazidi women located in internal displaced person (IDP) camps were interviewed. Of these 348, 139 females were survivors of ISIS captivity. Measures used included Kessler Psychological Distress Scale (K10), Patient Health Questionnaire (PHQ-9), Generalized Anxiety Disorder (GAD-7), and Harvard Trauma Questionnaire part IV (HTQ part IV).

**Results:**

Formerly enslaved Yazidi females showed a significantly higher prevalence of severe mental distress (97.1%; P < 0.001), more severe levels of depression (36.7%; P < 0.001) and general anxiety symptoms (37.4%; P < 0.001), greater rates of PTSD (90.6%; P < 0.001) and higher reported rates of suicidal ideation (38.1%; P < 0.001). Logistic regression analysis undertaken to examine the role of sociodemographic factors as predictors of the assessed mental health conditions. Amongst the formerly enslaved group, no such significance was found, however amongst the non-enslaved group, unemployment was found to statistically determine depression, generalized anxiety and PTSD. Specifically, women from the non-enslaved group who were unemployed were 2.5 times more likely to have depression, 3 times more likely to have generalized anxiety and 3.3 times more likely to have PTSD. Finally, amongst the non-enslaved group, those women with between 5 to 8 siblings were significantly less likely to have depression than those with fewer siblings.

**Conclusion:**

Rates of distress and trauma related symptomology were significantly higher amongst those with history of enslavement. Sociodemographic factors and duration of enslavement do not seem to predict mental disorders among enslaved females.

## Background

The Yazidis are Kurdish minority group living predominantly in the Northern Iraq, with a total worldwide population of 800,000 to 1,000,000 [1]. There are few written documents on Yezidi history or their belief system and instead are a highly oral culture with strong focus on song and storytelling. Due to their ancient faith that integrates elements of Islamic beliefs with Zoroastrianism and Mithraism, misunderstanding has meant that persecution has been a historical constant for this community [2, 3]. More recently, this community has suffered decades of discrimination and neglect by Saddam Hussein’s regime specifically and the Sunni extremists more broadly [4]. In August 2014, the terrorist organization known as the Islamic State of Iraq and Syria (ISIS) attacked began one of most deadliest genocides committed against the Yazidi homelands in northwestern Iraq [5]. Facing forced religious conversion, many were killed [6]. In space of two weeks ISIS fighters rounded the city of Sinjar and surrounding towns and villages driving Yazidis to flee or seek refuge on Mount of Sinjar. Those who could not run away quickly were either killed or abducted [6]. It is estimated that about 5500 Yazidis were killed and approximately 6000 endured some period under ISIS captivity [6, 6] In addition to the mass killing, Yazidi women in particular were subjected to murder, enslavement, torture, rape, sexual violence. Thousands were captured and sold into sexual slavery [6] and or forced to marry ISIS fighters with estimates that approximately 3537 women and girls were abducted and another 3200 Yezidi women and children are still being held in ISIS captivity [5].

There is now strong evidence to support the deleterious impact wars and atrocities such as genocides have on the survivor’s mental health and wellbeing [8]. Furthermore, research has shown that survivors of multiple traumas exhibit more higher rates and greater severity of posttraumatic stress disorder (PTSD) [9] and that victims of recurrent sexual assaults, such as was the case of ISIS slavery experiences can lead to development of severe levels of PTSD symptoms [9]. Indeed, the experience of rape and sexual violence have shown to be associated with serious mental health disorders [10]. The victims are more likely to suffer from PTSD and major depressive disorder (MDD) [11] with other sequalae including are other anxiety disorders, sexual dysfunction, dissociative disorders, and suicide attempts. [12].

To date, there has been a number of investigations that have demonstrated that the ISIS captivity and sexual slavery led to both immediate and long-term consequences for the mental health of the Yazidi women and girl survivors [13, 14]. Placing these experiences into the cultural and religious framework of the Yazidis, where any sexual contact or marriage outside the caste or with non-Yazidis is forbidden [1], it is clear that one of the most tragic outcomes being suicide may be related to the notion that some of these survivors killed themselves in order not to live with the supposed “dishonor” [1]. The impact of enslavement can be felt by the survivors through experiences such as witnessing of the death and murder of their spouses and children [11] and separation from their home environment leading to an interruption of their attachment to their homes, families and friends and leading to more traumatization [15].

Duhok is a northeast city in Iraq and hosts the largest proportion of internally displaced persons (IDPs) within 17 camps. These camps are overseen and managed by the Board of Relief and Humanitarian Affairs (BRHA), which has established the partnerships with core humanitarian agencies such as United Nations High Commissioner for Refugees (UNHCR), United Nations International Children's Emergency Fund (UNICEF) and German Corporation for International Cooperation (GIZ). It is estimated that the numbers of IDPs living in Duhok camps is approximately 168,183. According to BRHA report of January 2019, the majority of displaced families are originally from Sinjar District which represent more than half of the total IDPs in Duhok [16]. Currently, Yazidis Kurds represent 90.3% of the total population of camps inhabitants. Across all IDP camps in Duhok, 51% of adult camp inhabitants or 42,098 are adult females [16].

The UN organization, United Nations Fund for Population Activities (UNFPA) and the Directorate General of Health established the Duhok Survivors’ Centre to meet the physical and mental health needs of women and girls fleeing ISIS. The second center is located in Chamchamal city and is a project of the Jiyan Foundation for Human Rights, an international organization based in Germany with offices and programs in Iraq. This healing garden is a place of rest and security for women, children and their families who have experienced violence and persecution. These are the only two specialized facilities in Iraq that focus on helping women who have experienced gender-based violence. All ISIS female survivors entered these two centers for registration and/or therapy were invited to participate in our study during the period of January to May 2019.

With the previous research in mind, this cross-sectional study sought examine the impact of sexual slavery and captivity experienced by captured Yazidi females compared to those not enslaved on measures of trauma related mental health disorders and suicidal behaviours. Specifically, rates and symptom severity of major depressive disorder, generalized anxiety disorder, posttraumatic stress disorder, and suicide thoughts and behaviours were examined amongst Yazidi females with history of enslavement were compared to Yazidi females without such a history. Secondly, the role of sociodemographic factors as predictors of depression, generalized anxiety and PTSD amongst the two groups were analysed.

## Methods

### Study design and participants

A cross sectional comparative study design was undertaken between January and May 2019. Participants were 348 Yazidi women and girls who were living in IDP camps of Duhok city. The participants were divided into two groups which comprised of 139 Yazidi females who had survived ISIS slavery and 209 Yazidi females who had not experienced enslavement. Participants of the first group (formerly enslaved) were recruited from all new case registered at Duhok Survivors’ Centre and Jiyan Foundation for Human Rights during the period of January to May 2019. Inclusion criteria included being Yazidi background, female, 17 years age or older, held captive as an ISIS slave, living in the IDP camps in Duhok province in Kurdistan region of Iraq and an absence of cognitive impairment thereby allowing for the ability to comprehend and provide consent. A total of 150 Yazidi female survivors of ISIS captivity were asked to take part in this assessment. Of them, 11 refused to participate giving no reasons for refusal apart from their personal desire resulting in response rate of 92.7%.

Participants in the second group comprised of Yazidi females who had not experienced enslavement but were matched for all other inclusion criteria of enslavement group. Additionally, all participants (enslaved and non-enslaved) were recruited prior to receiving psychiatric treatment.

An attempt to approximate a random sample was undertaken when recruitment into the non-enslaved group. Using a simple random sampling method, the names of the 17 IDP camps managed by BRHA were assigned a number and one was selected, that being Khanky camp. The 17 camps are as follows: Bajid Kandala 1 and 2; Khanky; Qadya (Rawanga); Kabartu 1 and 2; Sharya; Dawidiya; Bersivy 1 and 2; Chamishko; Germawa; Esyan; Shekhan; Mamrashan; Mamilyan and Darkar. The number of adult females in this camp was 4,098. Next, using a systematic random sampling procedure, one from every 20 females was selected, resulting in 210 women invited to participate and all but one, agreed, resulting in response rate 99.5%.

Following, written informed consent, participants were interviewed by two experienced female clinical psychologists who were trained on the use of the study instruments. All measures were administered in either Arabic or Kurdish, as per the preference of the participant being interviewed. Data on socio-demographic characteristics was collected in addition to administration measures of psychological distress, major depressive disorder, generalized anxiety, and PTSD. All participants who were reporting symptom levels of moderate to severe and/or reported suicide risk were contacted by the psychiatrist in the two centres as a matter of urgent management.

Approval for the study was sought and obtained from the Scientific Committee of College of Medicine, University of Duhok and the Research Ethics Committee of Duhok Directorate General of health (reference number 12032019-2). Further, permission from the Directors of the Duhok Survivors’ Centre and Jiyan Foundation for Human Rights were sought and obtained.

### Study Instruments

#### Sociodemographic data

Measures of socio-demographic characteristics and enslavement data were collected. This namely included age, marital status, original and current address, number of siblings, current household members, education, occupation, family socio-economic status, length of enslavement (if any), and sexual assault/rape history.

#### Kessler psychological distress scale (K10)

The K10 is a 10-item questionnaire intended to yield a global measure of distress based on questions regarding anxiety and depressive symptoms that a person has experienced in the most recent 4 weeks period.[17] [18] Using a 5-point scale (1 = none of the time; 5 = all of the time), a total score of 25 or greater has been used to estimate prevalence of mental disorders. [18] This measure had a high internal reliability (Cronbach’s α = 0.908).

#### Patient health questionnaire (PHQ-9)

PHQ-9 is an self-report instrument used for criteria based diagnosis of depressive disorder.[19] It consists of 9 items derived from diagnostic and statistical manual of mental disorders (DSM-IV).[20] Participants are asked to consider over the last 2 weeks, how often they have been bothered by a list of nine problems, for example, “Little interest or pleasure in doing things”. Items are scored against a Likert scale ranging from 0 (Not at all) to 3 (Nearly every day) with summed total scores ranging from 0 to 27.[21] It can be used also to assess depression severity with categories as follows: ‘no depression’ (1–4), mild depression (5–9), moderate depression (10–14), moderately severe depression (11–19) and severe depression (20–27). Cronbach’s alpha in the present study sample was α = 0.837, indicating good reliability.

#### Generalized anxiety disorder 7-item (GAD-7) scale

GAD-7 a 7-item scale designed to assess symptoms of anxiety [20]. Participants are asked to how frequently over past two weeks they have been bothered by list of seven problems such as for example, “Feeling nervous, anxious, or on edge” [21]. Items are scored as: 0 = not at all, 1 = several days, 2 = more than half the days, 3 = nearly every day with summed total scores ranging from 0 to 21. Total GAD-7 scores are categorized minimal (0–4), mild (5–9), moderate (10–14) and severe (14–20). The GAD-7 is considered to be reliable to assess anxiety and the internal consistency for this study sample was moderate (α = 0.781).

#### Harvard trauma questionnaire (HTQ)

The HTQ is a widely used, cross-cultural self-report checklist assessing the occurrence and frequency of various types of traumatic events as well as PTSD symptomatology. It consists of four parts, however only part IV was included in the present study, being directly relevant to the assessment of PTSD. A score of 2.5 or above is considered to indicate a high probability of clinically significant PTSD symptomatology [22]. The HTQ has very good psychometric properties, including high test–retest reliability and internal consistency for part IV. Cronbach’ s alpha in the current study was high (α = 0.950).

### Statistical analysis

Statistical analyses were carried on using the IBM Statistical Package for Social Sciences (SPSS), version 22.0. [23] Comparisons were undertaken to examine the difference between the two groups (enslaved versus non-enslaved) across the severity categories for each mental health measures using Chi-square test of independence or Fisher’s exact test, as appropriate, or t-tests. To test the influence of socio-demographic characteristics and enslavement related variables on the mental health measures amongst the two participant groups (enslaved and non-enslaved), binary logistic regression was undertaken.

## Results

### Socio-demographic characteristics

Table 1 presents the socio-demographic characteristics of the two participant groups.

**Table 1:** Socio-demographic characteristics of the enslaved versus non-enslaved Yazidi females

	Total group (n = 348)		Formerly enslaved (n = 139)	Non-enslaved (n = 209)	Comparison	
	M (SD, range)		M (SD, range)	M (SD, range)	t	P
Age	31.9 (14.5, 17–96)		30.7 (13.6, 17–75)	32.8 (15.1, 17–96)	− 1.31	0.191
Years of education ^a^	2.13 (3.6, 0–12)		2.16 (3.3, 0–12)	2.12 (3.8, 0–12)	0.109	0.913
Months of enslavement	–		13.86 (16.06, 1–60)	–		
Comparison						
	N (%)		N (%)	N (%)	X^2^	P
Current Marital status					10.915	0.001*
Married	235 (67.5)		108 (77.7)	127 (60.8)		`
Unmarried	113 (32.5)		31 (22.3)	82 (39.2)		
Current Work Status					206.86	< 0.001**
Employed	172 (49.4%)		3 (2.2)	169 (80.9)		
Unemployed	176 (50.6%)		136 (97.8)	40 (19.1)		
Number of siblings					0.579	0.749
1–4 siblings	51 (14.8)		23 (16.5)	28 (13.6)		
5–8 siblings	192 (55.7)		76 (54.7)	116 (56.3)		
≥ 9 siblings	102 (29.6)		40 (28.8)	62 (30.1)		

M mean, SD Standard Deviation

The mean age of the females as a whole group was 31.9 (± 14.5) years. The mean age of the enslaved group was 30.7 years and (± 13.6) years and the non-enslaved average age was 32.8 (± 15.1) years. Both groups have minimal years of education which ranged for the group as a whole from 0 to 12 years, with average years of education amongst enslaved group 2.16 years compared to 2.12 years for the non-enslaved group. Marital status differed significantly between the two groups. In particular, while a majority of participants in both groups were married (77.7% enslaved and 60.8% of non-enslaved), there was a larger proportion of unmarried women amongst the non-enslaved group compared to the enslaved group (X^2^ = 10.92, p = 0.001). There was also a statistically significant difference between the two groups with respect to employment status, with only 2.2% of formerly enslaved women employed compared to 80.9% of non-enslaved women (X^2^ = 206.86, p < 0.001). Both groups were similar in number of siblings reported, ranging from 0 to 16 for the group as a total. Finally, the length of reported ISIS captivity for the enslaved group ranged from 1 to 60 months with a mean of 13.86 month.

### Clinical characteristics

Table 2 reports the rates of psychological distress levels, the frequency and severity categories of common trauma related mental health disorders and suicidal behaviors amongst enslaved versus non-enslaved Yazidi females.

**Table 2:** Psychological distress, mental health disorders and suicidal behaviours amongst enslaved versus non-enslaved Yazidi females

	Total group (n = 348) N (%)	Formerly enslaved (n = 139) N (%)	Non-enslaved slaves (n = 209) N (%)	χ2	P
Psychological Distress Levels (K10)				139.958	< 0.001**
None	105 (30.2)	8 (5.8)	97 (46.4)		
Mild	65 (18.7)	14 (10.1)	51 (24.4)		
Moderate	34 (9.8)	7 (5)	27 (12.9)		
Severe	144 (41.4)	110 (79.1)	34 (16.3)		
Depression (PHQ-9)				119.284	< 0.001**
Minimal or none	27 (7.8)	4 (2.9)	23 (11)		
Mild	27 (7.8)	12 (8.6)	87 (41.6)		
Moderate	87 (25)	25 (18)	62 (29.7)		
Moderately severe	80 (23)	47 (33.8)	33 (15.8)		
Severe	55 (15.8)	51 (36.7)	4 (1.9)		
Generalised Anxiety (GAD-7)				111.283	< 0.001**
None	60 (17.2)	9 (6.5)	51 (24.4)		
Mild	122 (35.1)	19 (13.7)	103 (49.3)		
Moderate	106 (30.5)	59 (42.4)	47 (22.5)		
Severe	60 (17.2)	52 (37.4)	8 (3.8)		
Probable PTSD (HTQ-IV)				87.355	< 0.001**
No	137 (39.4)	13 (9.4)	124 (59.3)		
Yes	211 (60.6)	126 (90.6)	85 (40.7)		
Suicide thoughts				19.166	< 0.001**
None	259 (74.4)	86 (61.9)	173 (82.8)		
Yes	89 (25.6)	53 (38.1)	36 (17.2)		
Suicide attempts				3.991	0.064
None	314 (90.2)	120 (86.3)	194 (92.8)		
Yes	34 (9.8)	19 (13.7)	15 (7.2)		
Method of suicide attempt				9.576	0.088
Drug ingestion	5 (18.5)	4 (28.6)	1 (7.7)		
Self- immolation	5 (18.5)	1 (7.1)	4 (30.8)		
Hanging	6 (22.2)	2 (14.3)	4 (30.8)		
Drowning	3 (11.1)	2 (14.3)	1 (7.7)		
Wrist cutting	4 (14.8)	4 (28.6)	0 (0)		
Electrocution	4 (14.8)	1 (7.1)	3 (23.1)		

There was a statistically significant difference found on psychological distress levels reported by the two group. Specifically, 79.1% of the formerly enslaved group reported severe distress levels compared with 46.4% of the non-enslaved group who reported no distress (p < 0.001). Significant differences were found amongst the two groups with respect to depression levels (p < 0.001). In particular, moderately severe (33.8%) and severe depression (36.7%) was reported more frequently by the enslaved group compared to the non-enslaved group who were more frequently reporting mild levels of depression (41.6%). A similar pattern was noted for generalized anxiety symptoms, where more severe levels were noted by the formerly enslaved group compared with non-enslaved group (p < 0.001). Notably, those who were formerly enslaved more frequently reported moderate (42.4%) and severe (37.4%) compared with mild anxiety symptoms being reported by 49.3% of the non-enslaved group. Probable PTSD was statistically more common amongst the enslaved group compared with non-enslaved group (90.6% versus 40.7%; p < 0.001). A statistically difference was noted for rates of self-reported suicidal thoughts which were more frequent amongst the formerly enslaved versus non-enslaved group (38.1% versus 17.2%; p < 0.001). Higher rates of self-reported suicidal attempts were noted for the enslaved group (13.7% versus 7.2%) but this was not statistically significant. Similarly, no statistical difference was noted for methods of suicide between the groups although some patterns were observed such as more frequently reported drug ingestion (28.6%) and wrist cutting (28.6%) amongst the formerly enslaved group and self-immolation (30.8%) and hanging (30.8%) amongst the non-enslaved group.

Table 3 reports the distribution of the number of probable mental health diagnoses amongst the participants in the two groups.

**Table 3:** Comorbidity of probable mental health disorders amongst the enslaved versus non-enslaved Yazidi females

Number of probable diagnoses	Total group (n = 348) N (%)	Formerly enslaved (n = 139) N (%)	Non-enslaved (n = 209) N (%)	χ2	P
Without diagnosis	72 (20.7)	7 (5)	65 (31.1)	34.562	< 0.001**
Single diagnostic category					
Only depression#	27 (7.8)	3 (2.2)	24 (11.5)	10.143	0.001*
Only GAD	6 (1.7)	0 (0)	6 (2.9)	4.060	0.085
Only PTSD	42 (12.1)	6 (4.3)	36 (17.2)	13.107	< 0.001**
Two diagnostic categories					
Depression and GAD	32 (9.2)	3 (2.2)	29 (13.9)	13.727	< 0.001**
Depression and PTSD	24 (6.9)	8 (5.8)	16 (7.7)	0.469	0.527
GAD and PTSD	6 (1.7)	3 (2.2)	3 (1.4)	0.257	0.686
Three diagnostic categories					
Depression, GAD, and PTSD	139 (39.9)	109 (78.4)	30 (14.4)	124.822	< 0.001**

Depression, Patient Health Questionnaire-Depression Module total score ≥ 10; GAD, Generalized Anxiety Disorder total score ≥ 10, PTSD, post-traumatic stress disorder mean score ≥ 2.5

Amongst the total sample, 72 participants (20.7%) did not report symptoms to indicate a probable mental health disorder and this was significantly more likely amongst the non-enslaved group than the formerly enslaved (31.1% versus 5%; p < 0.001). Participants in the non-enslaved group were more likely only one probable mental health disorder, namely either depression (11.5% versus 2.2%, p = 0.001) and PTSD (17.2% versus 4.3%; p < 0.001). Most notable was that 78.4% of the formerly enslaved group had 3 probable mental health disorders comorbidly (depression, GAD and PTSD) compared to 14.4% of the non-enslaved group (p < 0.001).

### Predictors of Probable Mental Health Disorders

Table 4 reports that odds ratios and 95% confidence intervals of socio-demographic characteristics as predictors for probable depression, GAD and PTSD by groups (formerly enslaved and non-enslaved).

**Table 4:** Odds ratios and 95% confidence intervals of socio-demographic characteristics as predictors for probable depression, GAD and PTSD by groups (formerly enslaved and non-enslaved)

	Formerly enslaved (n = 139)						Non-enslaved (n = 209)					
	Depression		GAD		PTSD		Depression		GAD		PTSD	
	Odds Ratio	95% CI	Odds Ratio	95% CI	Odds Ratio	95% CI	Odds Ratio	95% CI	Odds Ratio	95% CI	Odds Ratio	95% CI
Predictors												
Age	0.98	0.94–1.03	1.04	0.99–1.09	1.06	0.97–1.15	0.99	0.97–1.01	1.01	0.98–1.02	1.01	0.99–1.03
Education	0.87	0.72–1.05	1.02	0.86–1.22	1.07	0.86–1.32	0.98	0.89–1.08	0.93	0.84–1.04	0.91	0.81–1.01
Marital Status												
Married (ref)	(1)		(1)		(1)		(1)		(1)		(1)	
Unmarried	0.64	0.15–2.75	2.09	0.52–8.3	0.66	0.14–3.06	0.54	0.26–1.1	0.85	0.4–1.79	0.63	0.31–1.3
Employment status												
Employed (ref)	(1)		(1)		(1)		(1)		(1)		(1)	
Unemployed	6.56	0.4–105.95	2.83	0.18–42.41	3.96	0.21–71.96	2.54	1.01–6.36	3.05	1.18–7.91	3.32	1.25–8.85
Number of siblings												
1–4 siblings (ref)	(1)		(1)		(1)		(1)		(1)		(1)	
5–8 siblings	1.84	0.44–7.62	0.83	0.22–3.03	3.05	0.58–15.87	0.38	0.15–0.91	0.59	0.24–1.45	0.89	0.36–2.15
≥ 9 siblings	2.12	0.42–10.58	1.42	0.32–6.27	1.94	0.33–11.34	0.63	0.24–1.64	0.86	0.33–2.28	1.3	0.46–3.43
Length of enslavement	1.00	0.96–1.03	1.01	0.97–1.03	0.98	0.94–1.02	–	–	–	–	–	–

When considering the formerly enslaved Yazidi group, no significant association was found between any demographic characteristics including duration of enslavement with the any of measures of mental health disorders. With reference to the non-enslaved Yazidi females, those who were unemployed were over two times more likely to have depression as assessed by the PHQ-9 (OR = 2.54, 95% CI = 1.01–6.36; p < 0.05), while a minor relationship was found between depression and having 5–8 siblings (OR = 0.38, 95% CI = 0.15–0.91; p < 0.05). The impact of unemployment was even more pronounced on anxiety assessed by GAD-7 (OR = 3.05, 95% CI = 1.18–7.91; p < 0.05) and PTSD as measured by HTQ part IV (OR = 3.32, 95% CI = 1.25–8.85; p < 0.05).

## Discussion

In August 2014, the Yazidi population of Iraq and more specifically of Sinjar, were desolated by ISIS attacks. [6] These attacks resulted in approximately that 2.5% of the said population killed or abducted in the space of few days. [6] The atrocities were indiscriminating and executions of the most vulnerable being children, the elderly, and women as brutally common as those of the men who sought to defend their community. Females were enslaved, separated and forcibly shifted to different locations in ISIS controlled territorial dominion. Once enslaved, the girls and women were sold or gifted to ISIS fighters, subjected to prolonged sexual assaults, rape and physically tortured. [14] To date, it is estimated that more than 3000 Yazidi females remain in ISIS captivity. [24].

To the best of our knowledge, this is first large comparative study that sought to examine the mental health of formerly enslaved versus non-enslaved Yazidi females, prior to receiving any psychological intervention. Specifically, measures of psychological distress, depression, anxiety, and PTSD were examined amongst those females living in IDP camps located in Duhok, North Iraq. By far, the most notable finding was the significantly more frequent levels of severe psychological distress, severe depression and anxiety symptoms and higher rates of probable PTSD and self-reported suicidal thoughts noted amongst the enslaved group compared with the non-enslaved group. While the finding of higher rates of mental health disorders amongst those enslaved, of and in itself, is not surprising given the significant literature on the psychological sequalae of sexual assault, rape and torture [10], it is the first study to examine such rates in psychiatric treatment naïve participants, and thus revealing the true impact of such experiences. Our finding of that over 90% of formerly enslaved Yazidi females had probable PTSD is well above previously reported rates of 57% [14] but consistent with others.[13] Differences between sample locations may account for this. Specifically, in Kizilhan study [14], participants were recruited from those living in Germany, whereas ours were recruited from IDP camps in North Iraq. The possibility that being removed from the region where some ISIS fighters continue to live, may have reduced ongoing fear and this may have led to reduced symptomology cannot be discounted. On the other hand Ibrahim et al. noted rates of probable PTSD amongst the 65 formerly enslaved females surveyed to be between 98.5 and 100% dependent on the cut-off score utilised [13]. Although slightly higher than ours, possibly due to differences in sample size and measures utlised, these rates further demonstrate the compelling evidence of the relationship between exposure to prolonged sexual, physical and psychological torture and the development of PTSD [6]. Another finding of interest from our study was that rates of moderate and above depression for both groups were well above rates of moderate and above generalized anxiety. Over 88% of those females from the formerly enslaved group had at least moderate or greater levels of depression compared with 79.8% of said group demonstrating moderate or more levels of anxiety. Amongst the non-enslaved group, this difference was even more pronouce, with almost twice as many females in this group having at least moderate or greater depression than similar severity of anxiety (47.4% compared to 26.3%). This would indicate that depression is a frequent response to the experience of trauma, a finding that has also been noted in both general populations and refugees [25] and in sexual assaulted women from war affected populations [12]. The importance of treatment interventions that undertake a multifocal approach is even more necessitated by our finding of the high levels of comorbidity within the formerly enslaved females, with 78% having met the cut off scores for all three mental health scales assessed.

Measures of suicidal thoughts and attempts also differed between the two groups. In particular, our findings indicated that females from the formerly enslaved group were significantly more likely to have suicidal thoughts compared to the non-enslaved group but did not differ significantly with regards to self-reported rates of suicide attempts or methods. Previous research on suicide amongst refugee populations is conflicting with some indicating these populations are at elevated risk but others not [26]. Most relevant has been reports from organisations assisting in the rehabilitation of Yazidi females, which have indicated that these women are at increased risk of suicide attempts and completions but more rigorous investigations are needed [27]. In the past 4 years, efforts by the Yezidi community to undertake healing rituals in their holy city Lalish to assist in the rehabilitation of formerly enslaved females, is likely to have a positive impact on these tragic outcomes, but the distinct possibility of ongoing discrimination perpetrated within their own community continues to play a role their suicide behaviours [7].

When examining the role of socio-demographic predictors on mental health disorders amongst the two groups, unemployment was found to strongly predicted depression, generalized anxiety and PTSD in the non-enslaved group. Conversely, siblings, namely those with between 5 to 8 siblings, were less likely to have depression, within the non-enslaved group. These findings seem to support the notion, that ongoing contextual factors such as access to an income and social support, presumably what having a larger family may represent, are important factors in determining responses to traumatic experiences and this is consistent to other investigations [13]. Our finding that no socio-demographic factors were found to be predictive amongst the formerly enslaved group is also telling, providing further support for the harmful role that being enslaved and subjected to prolonged sexual assault has on mental health outcomes regardless of sociodemographic factors. Our findings are similar to Ibrahim et al. [13] where income was found to be have protective effect on PTSD and depression amongst the non-enslaved group, whereas only enslavement events found to significantly predict PTSD and depression in the former enslaved group.

Strengths of the current research include the initial recruitment of a relatively large, comparative samples, the collection of data by means of in-person interviews conducted by highly experienced female clinical psychologists who were able to undertake the interviews in either Arabic or Kurdish according the participants preference and the assessment of a broad range of demographic variables and other potential covariates. Several limitations of the research should also be noted. Firstly, despite significant efforts to obtain a representative sample of Yazidi females from both groups, assessing only those 17 years and older, precluded the representation of adolescent females. Secondly, the examination of depression, anxiety and PTSD, all common trauma related disorders, would have benefited with a measure of complex PTSD, which has been increasingly recognised as a mental health disorder following exposure to prolong and intense traumas. On the matter of assessment, while the mental health conditions were examined using commonly utilised cross-cultural tools, they were however self-report questionnaires rather than structured interview. The sample size of formerly enslaved Yazidi females, while larger than previous investigations, was small, hindered by the fact that access to psychiatric treatment naïve participants is difficult, with most either having already started therapeutic interventions and others are still in captivity.

## Conclusion

Exposure to enslavement has significant impact on mental health outcomes leading to greater rates of depression, generalized anxiety and PTSD. Our finding that amongst those formerly enslaved thoughts of suicide were significantly more frequent highlight the importance of urgent psychosocial rehabilitation. Efforts to develop and report on programs that aim to address this significant trauma within the unique cultural context of Yazidi community, should be the immediate and next steps.

## Data Availability

The data from this study cannot be shared in order to protect the anonymity of the participants.

## Abbreviations

BRHA: Board of Relief and Humanitarian Affairs
DSM: Diagnostic and Statistical Manual of Mental Disorders
GAD-7: Generalized Anxiety Disorder -7 Item Scale
GIZ: German Corporation for International Cooperation
HTQ: Harvard Trauma Questionnaire
IDP: Internally Displaced Person
ISIS: Islamic State of Iraq and Syria
K10: Kessler Psychological Distress Scale-10
MDD: Major Depressive Disorder
PHQ-9: Patient Health Questionnaire
PTSD: Posttraumatic Stress Disorder
UN: United Nations
UNFPA: United Nations Fund for Population Activities
UNHCR: United Nations High Commissioner for Refugees
UNICEF: United Nations International Children's Emergency Fund
